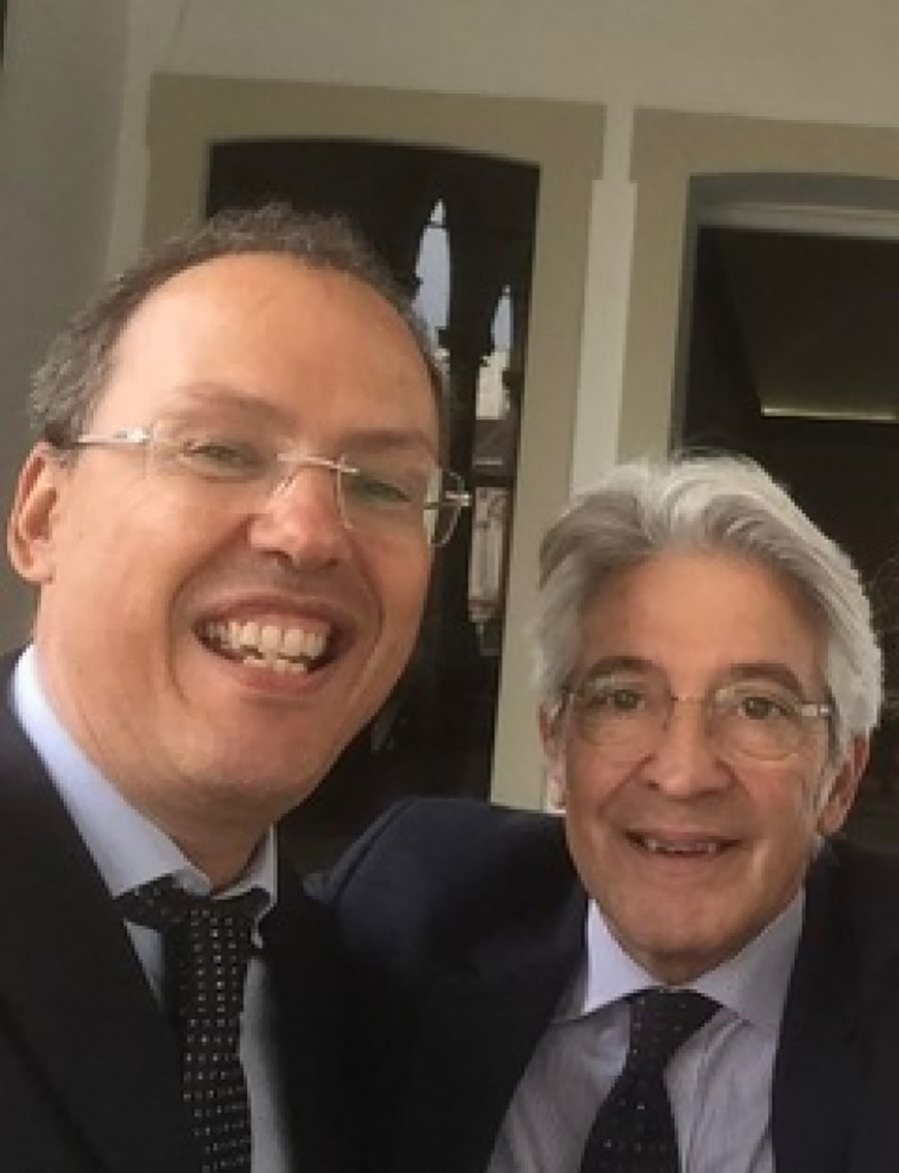# Roberto Copetti, MD (1954–2024)

**DOI:** 10.1186/s13089-024-00372-7

**Published:** 2024-03-26

**Authors:** Giovanni Volpicelli

**Affiliations:** Department of Medical and Surgical Science, Magna Græcia University, Catanzaro, Italy

One of those phone calls that you would never like to receive.

The first time that I met Roberto Copetti was in Torino during an international conference on Emergency Medicine. It was almost 20 years ago. At that moment I was publishing my first paper on lung ultrasound, a trial on more than 300 patients demonstrating the usefulness of B-lines compared to conventional tools in the diagnosis of pulmonary diseases in the emergency department. I attended the conference to listen to Roberto’s presentation, which aligned closely with the topic of my own research. I knew Roberto by his reputation as a pioneer of lung ultrasound, and I was particularly attracted by his way of presenting on this new tool. The hall was crowded, and I was standing in the back. His presentation concluded the session, and as always when he presented in congresses, at the end of his clear and erudite talk many colleagues and students gathered around him for questions and compliments. Although pressed, his immediate thought was to indicate and turn to me and, still using the microphone, said: “Hey, you colleague, I saw you listening, and I would like to ask you: why should we use chest radiography as a gold standard to validate lung ultrasound when we know well that sonography is superior?”. Clearly, he was interested in a discussion on this topic, overlooking the fact that we had never met before. I was there to learn, and he was in the position of teaching me rather than merely listening to my opinion.

This episode says all about who Roberto Copetti was. After that conference, my initial good impression of Roberto was confirmed during the long-time professional relationship and friendship that followed. He was humble, deeply passionate about his work, and notably at the forefront in many aspects of his field. He was excellent in the organization of his work and in transmitting his experience in emergency medicine to the younger generation. He was a great pioneer of lung ultrasound in emergency medicine, without self-celebration, and with solid knowledge. He was a master in lung ultrasound, the field that more than others linked us during these 20 years.

For all these reasons, once more, that phone call was one of those that I would never have liked to receive.

Bye Roberto

Prof. Giovanni Volpicelli

Editor-in-Chief of the Ultrasound Journal.